# Comparative effectiveness of combined ATFL and CFL repair versus ATFL‐only repair for severe chronic lateral ankle instability

**DOI:** 10.1002/jeo2.70345

**Published:** 2025-07-13

**Authors:** Kensei Yoshimoto, Masahiko Noguchi, Ayako Tominaga, Mitsuki Kumaki, Takumi Koseki, Ken Okazaki

**Affiliations:** ^1^ Department of Orthopedic Surgery Tokyo Women's Medical University Shinjuku‐ku Tokyo Japan; ^2^ Orthopaedic Foot and Ankle Center, Shiseikai Daini Hospital Setagaya‐ku Tokyo Japan

**Keywords:** anterior talofibular ligament, arthroscopic repair, calcaneofibular ligament, lateral ankle instability, ligament repair, recurrent ankle instability

## Abstract

**Purpose:**

Repairing only the anterior talofibular ligament (ATFL) remnant in severe chronic lateral ankle instability (CLAI) with a concurrent calcaneofibular ligament (CFL) injury can result in persistent instability, highlighting the need to include CFL repair. Thus, this study aimed to examine the effectiveness of combined CFL and ATFL repair in severe CLAI with a concurrent CFL injury.

**Methods:**

Eighty ankles from 77 patients with severe CLAI with a concurrent CFL injury who underwent lateral ankle ligament repair were retrospectively examined. Severe CLAI with a concurrent CFL injury was defined as an ATFL that exhibited no mechanical resistance to hook palpation during arthroscopy. Among them, 39 ankles from 38 patients underwent arthroscopic ATFL repair between 2018 and 2021 (ATFL‐only group), whereas 41 ankles from 39 patients underwent open ATFL and CFL repair between 2021 and 2024 (CFL‐repair group). Outcomes included recurrent ankle instability (respraining of the operated ankle following surgery) and Self‐Administered Foot Evaluation Questionnaire (SAFE‐Q) scores.

**Results:**

No significant differences were observed between patient demographics in the two groups. Overall, 16 (41.0%) cases of recurrent ankle instability were observed in the ATFL‐only group compared with 7 (17.1%) in the CFL‐repair group, showing a significant difference (*p* = 0.026). No significant differences were found in the postoperative SAFE‐Q scores between the two groups. Multivariate analysis adjusted for age, sex, body mass index, follow‐up periods and sports participation revealed that ATFL‐only repair was associated with a significantly higher risk of recurrent ankle instability compared with combined ATFL and CFL repair.

**Conclusion:**

Although no significant difference was observed in postoperative SAFE‐Q scores between the two groups, repairing ATFL along with CFL could be more effective in achieving stable ankle than ATFL‐only repair for severe CLAI with a concurrent CFL injury.

**Level of Evidence:**

Level Ⅳ.

**Abbreviations**
acAL: accessory anterolateral
AITFL: anteroinferior tibiofibular ligament
ATFL: anterior talofibular ligament
BMI: body mass index
CFL: calcaneofibular ligament
CLAI: chronic lateral ankle instability
OT: obscure tubercle
SAFE‐Q: Self‐Administered Foot Evaluation Questionnaire

## INTRODUCTION

Ankle sprains are among the most common injuries in sports involving the lower extremities. In the majority of cases, patients achieve full recovery through nonoperative treatment. Nonetheless, despite appropriate conservative management, approximately 20%–30% of patients experienced chronic lateral ankle instability (CLAI), leading to persistent symptoms [6, 18]. Poor management of recurrent ankle sprains and chronic instability may result in degenerative changes in the ankles over time [25]. Although nonoperative therapies remain the first‐line treatment, surgical intervention is warranted in the event of unsuccessful outcomes to prevent the progression of ankle osteoarthritis.

Many surgical approaches exist for stabilising the ankle. Anterior talofibular ligament (ATFL) repair, represented by the Broström procedure, is the most popular surgical method for CLAI [1, 38, 39]. Despite the generally favourable clinical outcomes following open or arthroscopic ATFL repair, postoperative recurrent ankle instability remains a significant problem [15, 26, 34, 35, 36, 37].

Whether the quality of the ATFL remnant influences postoperative clinical outcome scores after ATFL repair remains controversial [4, 7, 14, 24]. However, recent reports have demonstrated that the repair of poor ATFL remnant only resulted in recurrent ankle instability [15, 35]. More than 90% of cases with a poor ATFL remnant condition indicated severe CLAI with a high probability of a concurrent calcaneofibular ligament (CFL) injury [19]. Therefore, surgeons might need to consider repairing the CFL in cases of severe CLAI with a concurrent CFL injury. A recent biomechanical study reported ankle instability persistence only when the ATFL was repaired without including the injured CFL [12]. Therefore, this study aimed to investigate whether combined ATFL and CFL repair can reduce the incidence of postoperative ankle instability in severe CLAI with a concurrent CFL injury. It was hypothesised that combined ATFL and CFL repair can improve ankle stability and reduce the incidence of postoperative recurrent ankle instability compared with ATFL‐only repair. To the best of our knowledge, this study was the first study which compared the clinical outcomes of combined ATFL and CFL repair versus ATFL‐only repair for severe CLAI with a concurrent CFL injury.

## MATERIALS AND METHODS

### Subjects

**Figure 1:**
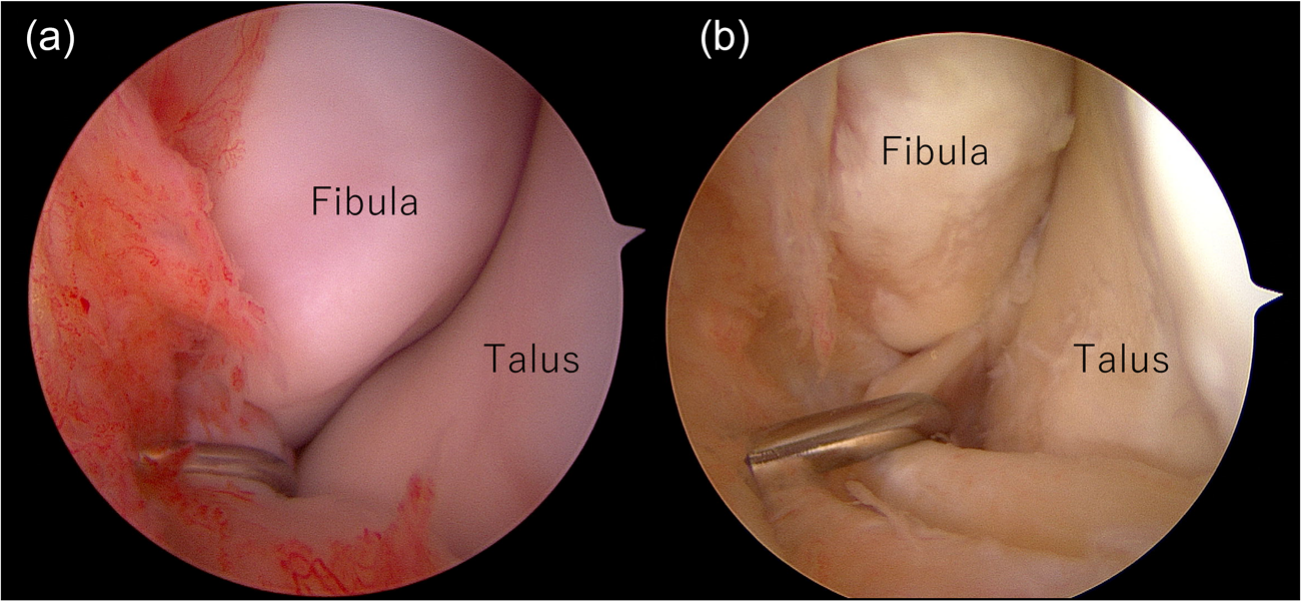
Arthroscopic classification of chronic anterior talofibular ligament (ATFL) injury. (a) Not severe chronic lateral ankle instability (CLAI): normal ATFL thickness with adequate mechanical resistance. (b) Severe CLAI: thin or absent ATFL without mechanical resistance.

**Figure 2:**
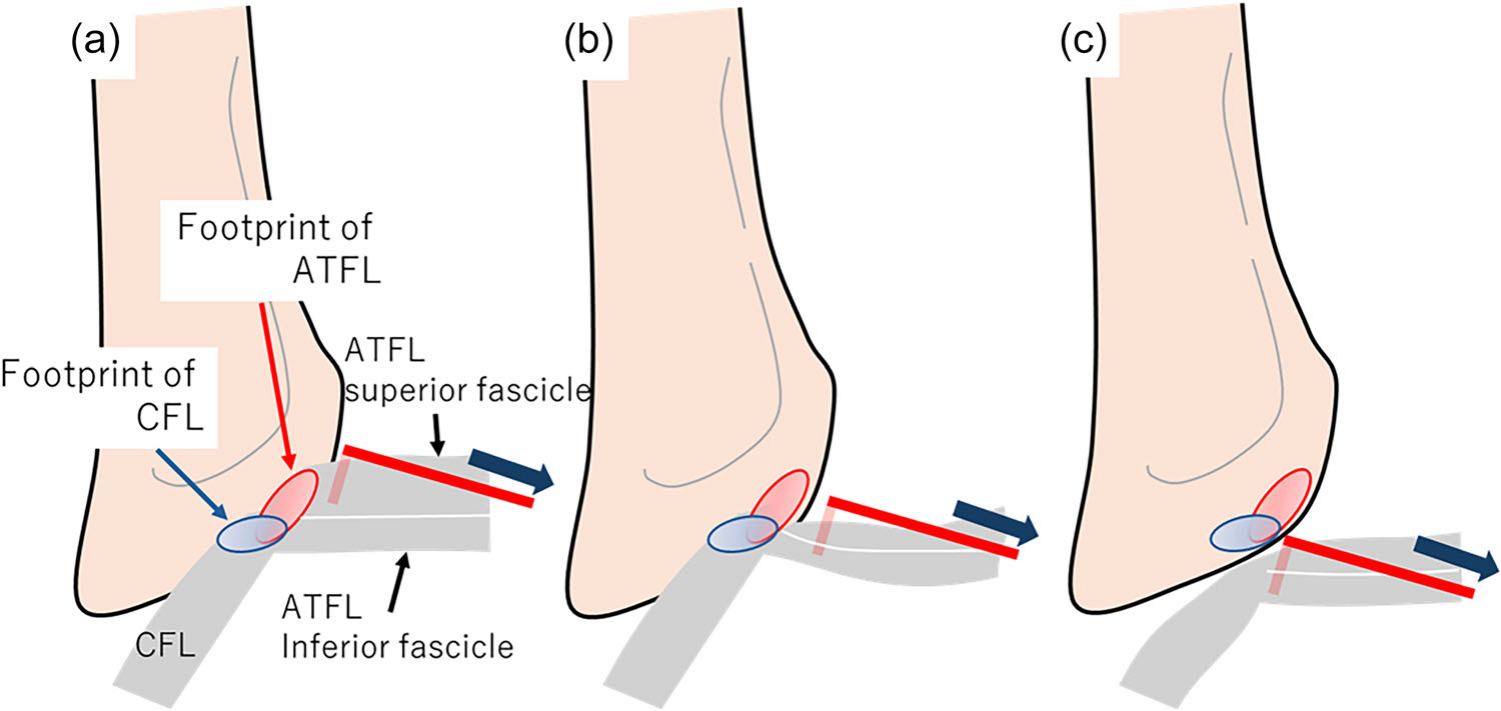
Intact ATFL and CFL can demonstrate adequate mechanical resistance by hook palpation (a). Because the footprints of the ATFL inferior fascicle and the CFL were interconnected, an injury limited to the ATFL superior fascicle, with an intact ATFL inferior fascicle and CFL, may still demonstrate adequate mechanical resistance by hook palpation (b). However, injuries to both the ATFL and CFL will not generate mechanical resistance (c). ATFL, anterior talofibular ligament; CFL, calcaneofibular ligament.

This study was approved by the institutional review board (IRB no. 130). Between 2018 and 2024, 262 ankles from 251 patients underwent lateral ankle ligament repair for CLAI. Surgery was indicated for unbearable pain and symptoms of instability that did not respond to nonoperative care [34]. Ankle instability was evaluated based on anterior drawer and talar tilt laxity, which were judged pathological when a ‘nonstop’ sign was detected [7, 34]. Among them, 105 ankles from 99 patients diagnosed with severe CLAI with a concurrent CFL injury were included in this study (Figure 1). Severe CLAI with a concurrent CFL injury was diagnosed through arthroscopy, which revealed that the ATFL did not exhibit mechanical resistance by hook palpation. Because the footprints of the ATFL inferior fascicle and CFL were interconnected [3, 32], it was anticipated that injuries to both ligaments would result in no mechanical resistance by hook palpation (Figure 2c). Intra‐ and interobserver reproducibility for this arthroscopic diagnosis method showed excellent agreement [35]. None of the patients exhibited ligamentous laxity associated with collagen disorders. A total of 22 patients (25 ankles) were excluded because of short (<1 year) follow‐up period (10 ankles from nine patients), obvious cavovarus deformity (one ankle from one patient), ankle osteoarthritis (Stage ≥ 2) based on the Takakura classification [27] (four ankles from four patients), previous foot and ankle surgery (six ankles from five patients), and neuromuscular disorders (four ankle from three patient). Finally, 80 ankles from 77 patients were included. Of this total number, 39 ankles from 38 patients underwent arthroscopic ATFL repair between 2018 and 2021 (ATFL‐only group), and 41 ankles from 39 patients were subjected to open ATFL and CFL repair between 2021 and 2024 (CFL‐repair group).

All the surgeries were performed by the same orthopedic surgeon. To evaluate the lateral ankle ligament, the surgeon performed an arthroscopic examination using a 2.7‐mm, 30‐degree arthroscope. Two portals were used during the surgery, namely, a viewing portal (medial midline portal) [28] and a working portal (accessory anterolateral [acAL]). ATFL remnant quality was assessed by hook palpation [35].

### ATFL‐only repair

**Figure 3:**
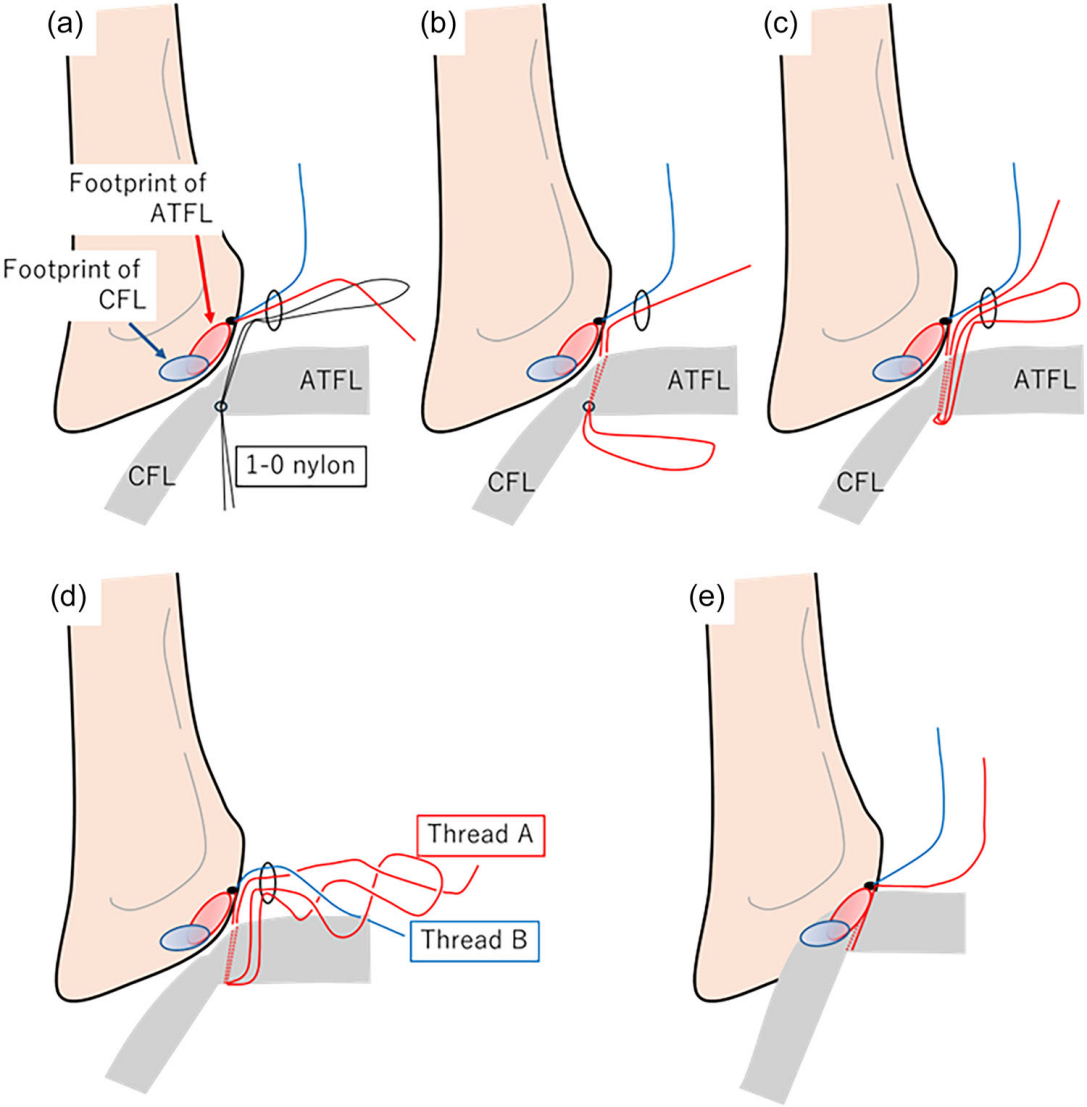
An 18‐G hollow needle with a 1–0 nylon thread was inserted through the fibula tip to penetrate the inferior bundle of the anterior talofibular ligaments after placing a suture anchor at the inferior edge of the anteroinferior tibiofibular ligament. The nylon loop was retrieved through the accessory anterolateral portal. One limb of the suture anchor was then passed through the nylon loop (a), and the opposite end of the nylon loop was withdrawn percutaneously (b). Subcutaneous passage of the suture loop through the accessory anterolateral portal was facilitated (c). The loop was twisted to guide thread B and then twisted again to guide thread A (d). Thread A was pulled lightly, whereas thread B was pulled firmly to reattach the footprint of the lateral ankle ligament. Finally, a square knot, followed by two granny knots, was tied using a knot pusher (e).

Arthroscopic ATFL repair was performed following a previously described procedure [35]. A suture anchor was placed at the inferior edge of the anteroinferior tibiofibular ligament (AITFL) [16] via the acAL portal. One limb of the suture anchor penetrated the inferior bundle of the ATFL [9]. Then, the ATFL was reattached to the footprint using the modified lasso loop stitch technique (Figure 3) [16].

### Combined ATFL and CFL repair

**Figure 4:**
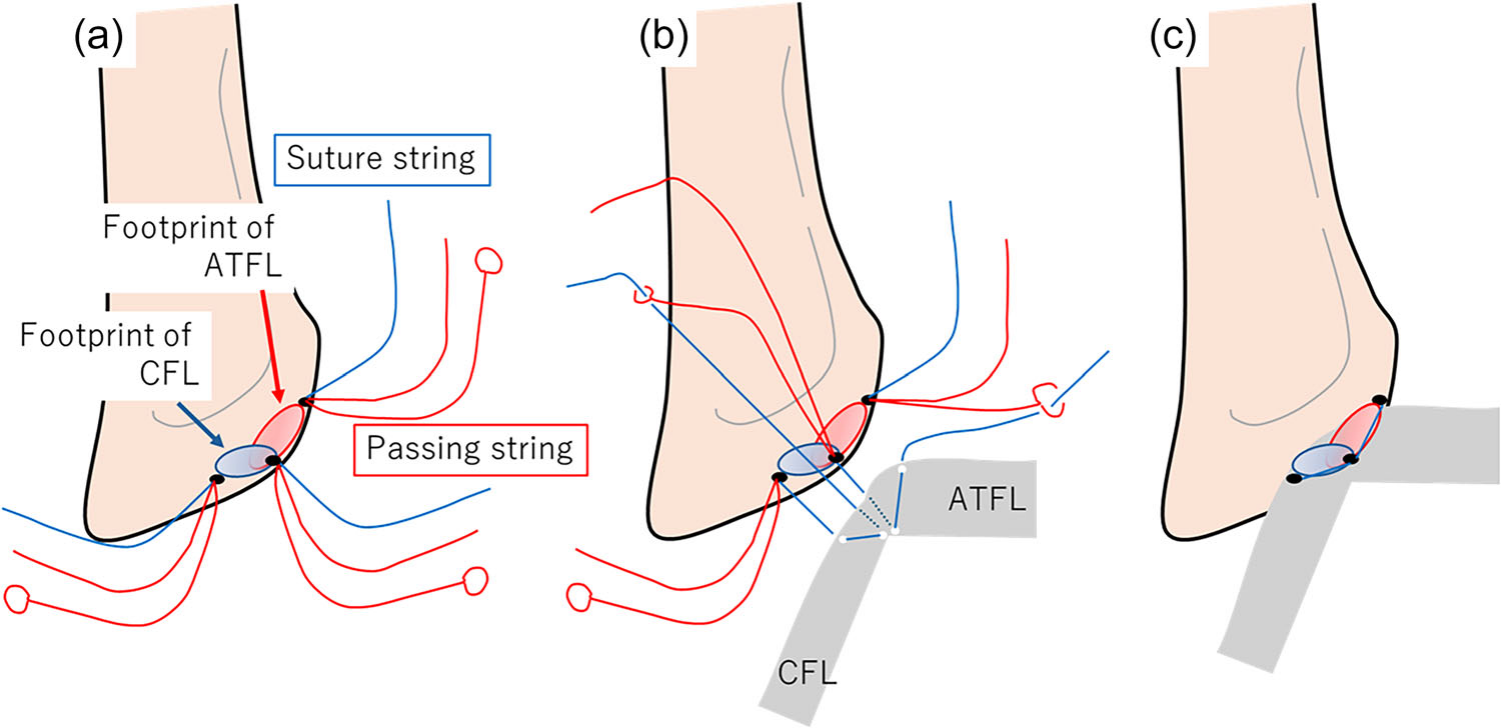
DEX Knotless FiberTak® suture anchor (Arthrex, Naples, FL, USA) was inserted into the superior edge of the ATFL attachment, OT, and posterior edge of the CFL attachment (a). The suture string inserted at the posterior edge of the CFL attachment threaded through the CFL and capsule complex and was passed into the small circle of a passing string inserted at the OT. The suture string inserted at the OT threaded through the ATFL and capsule complex was passed into the small circle of a passing string inserted at the superior edge of the ATFL attachment (b). The other passing string was drawn to attach the ATFL and CFL to the lateral malleolus (c). ATFL, anterior talofibular ligament; CFL, calcaneofibular ligament; OT, obscure tubercle.

Open repair of both the ATFL and CFL was performed through a 2‐cm skin incision along the anterior border of the fibula, centred over the obscure tubercle (OT) [16]. The capsule–ligament complex was incised from the site just distal to the attachment of the AITFL to the posterior edge of the CFL along the anterior border of the lateral malleolus without separating the capsules and ligaments [11]. Afterward, the DEX Knotless FiberTak® suture anchor (Arthrex, Naples, FL, USA) was inserted into the superior edge of the ATFL attachment, OT, and posterior edge of the CFL attachment (Figure 4a). This suture anchor involved one suture string and two passing strings. It features a self‐locking system, and the suture string can be passed through the locking mechanism using the passing strings. This enables precise tension control without the need for a knot [9], thereby reducing the risk of knot‐related pain. The suture string inserted at the posterior edge of the CFL attachment threaded through the CFL and capsule complex while protecting the peroneal tendon and was passed into the small circle of a passing string inserted at the OT. The suture string inserted at the OT threaded through the ATFL and capsule complex was passed into the small circle of a passing string inserted at the superior edge of the ATFL attachment (Figure 4b). Another passing string was drawn to attach the ATFL and CFL to the lateral malleolus (Figure 4c).

### Postoperative care

All patients were allowed full weight‐bearing from Day 1 after surgery with cast immobilisation (until 2022) or elastic ankle strap (from 2023). The cast was removed 1 week after surgery, and an elastic ankle strap was used 4 weeks after surgery. After cast removal, the patients engaged in exercises under the supervision of a physical therapist to restore muscle strength and range of motion in the affected ankle. They were deemed fit to resume full sports activities if they demonstrated normal ankle function during sport‐specific drills for at least 5 weeks after surgery.

### Evaluation of outcomes

The primary outcome was recurrent ankle instability. During their latest visit, patients were inquired if their surgically treated ankle had resprained postoperatively. Those who answered affirmatively were diagnosed with recurrent ankle instability [34, 35, 36]. To assess secondary outcomes, the Self‐Administered Foot Evaluation Questionnaire (SAFE‐Q) [22, 23] was used before surgery and at the latest visit after surgery.

### Data analysis

Statistical analysis was performed with JMP pro 17.0 software (SAS Institute, Cary, NC, USA). The differences between the two groups were evaluated using Fisher's exact probability test and Mann–Whitney *U* test for categorical and continuous variables, respectively. Multivariate logistic regression analysis adjusted for patients' demographics was performed to determine whether CFL repair could truly reduce recurrent ankle instability. Values of *p* < 0.05 were regarded as significant.

## RESULTS

No significant differences were found in patients' demographics and preoperative SAFE‐Q between the two groups (Table 1).

**Table 1:** Comparison of patients' demographics in ATFL‐only and CFL‐repair.

	ATFL‐only (39 ankles)	CFL‐repair (41 ankles)	*p* value
Age	41.7 ± 15.8	43.4 ± 14.6	0.482
Sex (male/female)	17/22	25/16	0.091
BMI	23.5 ± 3.2	24.6 ± 3.9	0.196
Follow‐up periods (month)	18.9 ± 7.7	16.6 ± 6.8	0.221
Sports participation (yes/no)	14/25	16/25	0.820
Preoperative SAFE‐Q			
Pain related	66.3 ± 24.5	66.0 ± 19.1	0.612
Physical functioning	78.0 ± 19.0	81.3 ± 16.1	0.551
Social functioning	72.5 ± 27.2	75.8 ± 25.5	0.653
Shoe related	79.2 ± 18.4	72.2 ± 25.9	0.385
General health	71.7 ± 23.2	70.8 ± 23.7	0.968

*Note*: Values are presented as mean ± standard deviation.

Abbreviations: ATFL, anterior talofibular ligament; BMI, body mass index; CFL, calcaneofibular ligament; SAFE‐Q, Self‐Administered Foot Evaluation Questionnaire.

*p < 0.05.

A total of 16 (41.0%) cases of recurrent ankle instability were observed in the ATFL‐only group and 7 (17.1%) in the CFL‐repair group, showing a significant difference (*p* = 0.026). Among them, two patients in the ATFL‐only group underwent revision surgery for recurrent ankle instability, whereas no patients in the CFL‐repair group did revision surgery. In addition, compared with the ATFL‐only repair group, one patient in the CFL‐repair group had a wound infection. Other surgical complications such as nerve, vascular, and tendon injuries did not occur. No significant difference was found (*p* > 0.05) in the postoperative SAFE‐Q scores between the two groups (Table 2).

**Table 2:** Comparison of postoperative outcomes and multivariate analysis adjusted for age, sex, body mass index, follow‐up period and sports participation.

	ATFL‐only (39 ankles)	CFL‐repair (41 ankles)	*p* value	Relative risk of ATFL‐only repair	*p* value
Recurrent instability (ankles)	16 (41.0%)	7 (17.1%)	0.026*	4.6	0.0180
Complications (ankles)	0	1	1		
Revision surgery (ankles)	2	0	0.235		
Postoperative SAFE‐Q					
Pain related	91.4 ± 14.0	92.4 ± 8.1	0.525		
Physical functioning	93.9 ± 8.5	96.3 ± 5.4	0.267		
Social functioning	95.7 ± 7.7	97.3 ± 4.5	0.681		
Shoe related	93.2 ± 12.5	88.2 ± 15.3	0.139		
General health	94.7 ± 11.0	93.7 ± 8.6	0.236		

*Note*: Values are presented as mean ± standard deviation.

Abbreviations: ATFL, anterior talofibular ligament; CFL, calcaneofibular ligament; SAFE‐Q, Self‐Administered Foot Evaluation Questionnaire.

* *p* < 0.05.

Multivariate analysis adjusted for age, sex, BMI, follow‐up periods, and sports participation also revealed that ATFL‐only repair had a significantly higher risk of recurrent ankle instability than combined ATFL and CFL repair (Table 2).

## DISCUSSION

The most crucial finding of this study was the low incidence of recurrent ankle instability following the combined ATFL and CFL repair compared with the ATFL‐only repair for severe CLAI with a concurrent CFL injury. This finding indicates that the repair of both CFL and ATFL for severe CLAI can lead to a more stabilised ankle joint.

Although long‐term studies of ATFL repair without CFL repair have shown good clinical outcomes [13, 21], an increasing number of biomechanical studies have highlighted the importance of the CFL. Hunt et al. [8] reported that the CFL plays a more significant role than the ATFL in ankle joint stability during load‐bearing inversion conditions. In addition, Larkins et al. [12] revealed that persistent ankle instability occurred when the ATFL was repaired without concurrently treating the injured CFL. Similarly, ATFL repair without addressing the injured CFL did not restore a normal strain pattern of the CFL [29]. Luthfi et al. [15] showed that repairing only the ATFL, while neglecting a CFL injury, may result in recurrent instability. These studies highlight the importance of the CFL in ankle stability.

A recent study using oblique CFL views on MRI demonstrated that patients with CLAI had a high rate of CFL injuries [20], and several studies have demonstrated feasible arthroscopic CFL repair [5, 33]. These studies on CFL injuries suggest that concern about the need for CFL repair is growing. However, it remains unclear how surgeons determine whether CFL repair is necessary. Nakasa et al. [19] determined the need for CFL repair using intraoperative stress radiography after ATFL repair and showed this procedure was effective. In the present study, the need for CFL repair was assessed by testing the mechanical resistance of the ATFL using hook palpation. The footprints of the ATFL inferior fascicle and CFL were interconnected [3, 32]. Therefore, if only the ATFL superior fascicle was injured and the ATFL inferior fascicle and CFL were intact, the ATFL inferior fascicle exhibited adequate mechanical resistance by hook palpation (Figure 2b). However, if both the ATFL and CFL were injured, no mechanical resistance could be noted. (Figure 2c). The finding that stable ankle function could be achieved through CFL repair in this study suggests that our procedure could accurately diagnose CFL injury.

Ko et al. [10] reported that combined ATFL and CFL repair did not show any superiority in clinical and radiographic outcomes by stress radiography compared with ATFL‐only repair. However, in the present study, combined ATFL and CFL repair reduced the incidence of recurrent ankle instability compared with ATFL‐only repair. These differences can be ascribed to the differences in the inclusion criteria: Ko et al. [10] included patients with CLAI without considering the severity of CLAI, whereas the present study enroled only patients with severe CLAI with a concurrent CFL injury. These findings indicated that ATFL‐only repair is insufficient for patients with severe CLAI with a concurrent CFL injury, thus necessitating CFL repair in such cases. On the other hand, this study showed no significant difference in postoperative SAFE‐Q scores between the ATFL‐only and CFL‐repair groups. However, since this was a short‐term follow‐up study, long‐term observation of patients with recurrent ankle instability might reveal a deterioration in SAFE‐Q.

This study has some limitations. First, instability was assessed in a qualitative manner, without any quantitative measurements such as stress radiography. However, stress radiography was deemed unnecessary to evaluate CLAI laxity because of a high false‐negative rate [31], and most surgeons do not often rely on it [17]. Second, the condition of CFL was not evaluated preoperatively. Because this study aimed to evaluate the effectiveness of CFL repair, CFL injuries should be examined on MRI or ultrasonography. Although accurate diagnostic methods and classification of chronic CFL injuries have not been established [2, 30], the oblique view of the CFL on MRI may be useful for diagnosing CFL abnormalities [20]. Third, the cause for recurrent ankle sprain after surgery was not recorded. If the recurrent ankle sprain was due to an accidental external factor, it likely wasn't related to the surgical technique. Fourth, different surgical techniques were used: ATFL‐only repairs were done arthroscopically, whereas CFL repairs were done via open surgery. Lastly, only short‐term outcomes were recorded.

## CONCLUSION

Considering both ATFL and CFL are susceptible to injuries in severe CLAI, their repair was more effective than ATFL‐only repair to achieve a more stable ankle. Although no significant difference was observed in postoperative SAFE‐Q scores between the two groups, the recurrence rate of ankle instability was lower in the combined ATFL and CFL repair group than in the ATFL‐only repair group. These findings underscore the importance of treating both ligaments to achieve better postoperative stability. The significance of this study lies in its potential to influence surgical practices and improve the quality of life of patients suffering from severe CLAI with a concurrent CFL injury.

## AUTHOR CONTRIBUTIONS

Kensei Yoshimoto contributed to the conception and design of the manuscript, decision regarding the treatment protocol, acquisition of data, and manuscript preparation. Mitsuki kumaki, Takumi Koseki and Ayako Tominaga contributed to the decision regarding the treatment protocol, the acquisition of data, and participated in the surgery. Masahiko Noguchi and Ken Okazaki made valuable suggestions regarding the design and conception of the study. All authors critically reviewed the manuscript, approved the final version of the manuscript, and have agreed to be accountable for all aspects of the work in ensuring that questions related to the accuracy or integrity of any part of the work are appropriately investigated and resolved.

## CONFLICT OF INTEREST STATEMENT

The authors declare no conflicts of interest.

## ETHICS STATEMENT

This study was approved by the institutional review board at Shiseikai Daini Hospital (IRB number: 130). Informed consent was obtained from all patients.

## Data Availability

The data that support the findings of this study are available on request from the corresponding author. The data are not publicly available due to privacy or ethical restrictions.
